# Minimally Processed Vegetables in Brazil: An Overview of Marketing, Processing, and Microbiological Aspects

**DOI:** 10.3390/foods12112259

**Published:** 2023-06-03

**Authors:** Jéssica A. F. F. Finger, Isabela M. Santos, Guilherme A. Silva, Mariana C. Bernardino, Uelinton M. Pinto, Daniele F. Maffei

**Affiliations:** 1 Department of Food and Experimental Nutrition, Faculty of Pharmaceutical Sciences, University of Sao Paulo, Sao Paulo 05508-000, SP, Brazil; jessicaaragao@hotmail.com (J.A.F.F.F.); uelintonpinto@usp.br (U.M.P.); 2 Food Research Center (FoRC-CEPID), Sao Paulo 05508-080, SP, Brazil; 3 Department of Agri-Food Industry, Food and Nutrition, “Luiz de Queiroz” College of Agriculture, University of Sao Paulo, Piracicaba 13418-900, SP, Brazil; isabela_santos@usp.br; 4 Department of Nutrition, Faculty of Public Health, University of Sao Paulo, Sao Paulo 01246-904, SP, Brazil; guilhermesilva1@usp.br (G.A.S.); marianabernardino15@usp.br (M.C.B.)

**Keywords:** fresh-cut vegetables, foodborne illness, microbiological safety, minimum processing, fresh produce

## Abstract

The global demand for minimally processed vegetables (MPVs) has grown, driven by changes in the population’s lifestyle. MPVs are fresh vegetables that undergo several processing steps, resulting in ready-to-eat products, providing convenience for consumers and food companies. Among the processing steps, washing–disinfection plays an important role in reducing the microbial load and eliminating pathogens that may be present. However, poor hygiene practices can jeopardize the microbiological quality and safety of these products, thereby posing potential risks to consumer health. This study provides an overview of minimally processed vegetables (MPVs), with a specific focus on the Brazilian market. It includes information on the pricing of fresh vegetables and MPVs, as well as an examination of the various processing steps involved, and the microbiological aspects associated with MPVs. Data on the occurrence of hygiene indicators and pathogenic microorganisms in these products are presented. The focus of most studies has been on the detection of *Escherichia coli*, *Salmonella* spp., and *Listeria monocytogenes*, with prevalence rates ranging from 0.7% to 100%, 0.6% to 26.7%, and 0.2% to 33.3%, respectively. Foodborne outbreaks associated with the consumption of fresh vegetables in Brazil between 2000 and 2021 were also addressed. Although there is no information about whether these vegetables were consumed as fresh vegetables or MPVs, these data highlight the need for control measures to guarantee products with quality and safety to consumers.

## 1. Introduction

Regular consumption of vegetables plays an important role in human nutrition, due to their vitamins, minerals, and dietary fiber content [1,2,3,4,5]. The search for a healthy diet by the population has resulted in an increase in the demand for vegetables, including minimally processed vegetables (MPVs) [2,6,7,8].

In the present context, the term minimally processed refers to the use of one or more methods, techniques, or procedures to transform plant-derived foods into ready-to-eat (RTE) or ready-to-cook (RTC) products with an extended shelf life while maintaining the same nutritional and organoleptic (sensory) quality of fresh vegetables [6,9,10]. In general, MPVs can have a shelf life ranging from a few days to two weeks, depending on several factors, such as the type and quality of fresh vegetables, processing method, type of packaging, storage conditions, and the presence of spoilage microorganisms [9]. When performed in accordance with good manufacturing practices, minimal processing delays nutrient loss and undesirable changes in texture, color, flavor, and aroma of vegetables, apart from microbial spoilage [11]. This holds significant importance, as these changes can lead to a decreased shelf life of the products and increase the likelihood of rejection by consumers and markets [12]. 

A wide variety of vegetables can be processed into MPVs, including leafy greens (e.g., arugula, lettuce, and spinach), cruciferous vegetables (e.g., broccoli and cauliflower), root vegetables (e.g., carrots and beets), and cucumbers, among others. These MPVs products are often sold as salads or snacks. In other words, the possibilities are endless, and the choice of vegetables depends on factors such as availability, demand, and consumer preferences. The market for these products has grown in Brazil, reflected by the increasing presence of these products in supermarkets and grocery stores across the country. Studies have shown that MPVs are sought by a range of consumers, mainly individuals with high levels of education and income, who are primarily attracted to the convenience offered by these products [13,14]. 

The expansion of fast-food chains, restaurants, and meal-producing companies also contributes to the increase in demand for MPVs [15,16]. Minimal processing offers consumers and/or companies the advantage of obtaining fresh vegetables with good quality, providing convenience and practicality while enabling producers to add value to their products [2,5,7]. Moreover, these products contribute to reducing food waste, since they are sold in customized portions, packaged, and stored under conditions that help preserve their freshness and extend their shelf life, requiring less preparation time in households when compared to whole vegetables.

Despite the advantages associated with MPVs, studies carried out worldwide have detected the presence of pathogenic microorganisms in these products, while epidemiological data from some countries have shown an association between the consumption of vegetables (including MPVs) and foodborne outbreaks, as discussed in the “Microbiological quality and safety of MPVs” section. Fresh vegetables are typically cultivated in open fields and are susceptible to pre- and post-harvest contamination. Minimal processing can also contribute to contamination through poor hygiene or cross-contamination that can occur during washing and other steps [16,17,18,19,20,21,22]. 

While numerous studies on MPVs exist in the literature, there is a notable gap when it comes to comparing them to their fresh counterparts, particularly in terms of market aspects, including a comparative price analysis. Furthermore, there is limited research addressing the specific processing characteristics involved in MPVs production. Moreover, the available review papers on their microbiological aspects focus on pathogenic microorganisms, disregarding the importance of studying the occurrence of hygiene indicators, which is a critical aspect of an RTE/RTC product.

The aim of this review is to provide an overview of MPVs, focusing on the Brazilian market, processing steps, and microbiological aspects. Data on the occurrence of hygiene indicators and pathogenic microorganisms in these products, as well as foodborne outbreaks associated with the consumption of fresh vegetables, are also presented. Although this study focuses on Brazilian data, information from other countries was also incorporated, mainly in the microbiological topic, to enable a comparison of the quality, safety, and microbiological criteria adopted for MPVs.

## 2. Market of MPVs in Brazil

In Brazil, the market of MPVs emerged in the mid-1970s with the expansion of fast-food chains in the southeastern region of the country, following a trend in the United States, where the market of these products started in the 1930s [15,16]. Currently, the increase in demand for these products seems to be a worldwide trend, resulting from the social, political, and economic changes that have changed habits and lifestyles [6,16,23]. 

The MPVs market has grown in Brazil over the past decades, driven by a lifestyle characterized by a reduced time for food preparation, as well as an increasing consumer demand for fresh and healthier products [16,24]. The presence of MPVs in supermarkets and grocery stores is steadily growing, particularly in large urban centers. A recent study conducted by Costa et al. [25] identified a total of 39 brands of MPVs being sold in the four most populous capitals located across different Brazilian regions (Northeast, Midwest, South, and Southeast). Most of these brands (20; 51.3%) were found in the southeastern region; four were found in more than one region, and none of them were found in all regions. However, the authors observed that MPVs were not available in any of the establishments visited in the capital selected for the North region. Apart from being sold in supermarkets and grocery stores, these products have gained popularity among industrial kitchens, caterers, hospitals, and hotels, which look for convenience combined with a reduction in workforce, less waste generation, and faster preparation of meals [26].

In retail settings, MPVs are typically displayed on refrigerated shelves, often positioned in close proximity to fresh vegetables. However, it is worth noting that in many Brazilian establishments, fresh vegetables are commonly sold without refrigeration. Among the marketing strategies aimed at promoting these products, it is possible to highlight the use of distinctive packaging that emphasizes their freshness, along with labeling that communicates their sanitized status and the convenience they offer for immediate consumption [15]. Furthermore, some MPVs producers opt to conduct product tastings at retail, as well as distribute samples to food services and fast-food chains, as a strategy to introduce these products to potential commercial buyers.

Regarding costs, MPVs generally tend to be more expensive to consumers in comparison to fresh vegetables. This is expected because the processing and storage of MPVs incur additional costs that are passed on to the products. Nevertheless, to our knowledge, no Brazilian studies have conducted a market comparison of the price of these products. Therefore, the team of researchers of the current study visited six supermarket chains and two farmer markets in the city of Sao Paulo, southeastern Brazil, to gather the relevant data, as presented in Table 1. The range of available fresh vegetables in the visited markets was broader compared to MPVs. However, to fulfill the purpose of providing a price comparison for the same vegetables in both formats, the table includes only samples that allow for a direct comparison per 100 g of product. As predicted, MPVs were found to be more expensive, with a difference in price ranging from 142.8% to 803.4% compared to fresh vegetables. 

High prices are identified as one of the most limiting factors for MPVs purchases among Brazilian consumers, as shown in previous studies. Sato et al. [27] conducted a survey with 42 individuals in the city of Sao Paulo, and 52% of the participants cited high prices as the primary reason for not purchasing MPVs. Similarly, Perez et al. [13] conducted a survey with 246 individuals in the city of Belo Horizonte, Minas Gerais state, and found that high prices were indicated by 31.9% of participants as the main limiting factor for purchasing MPVs. More recently, Finger et al. [14] conducted an online survey with 1510 consumers in Brazil and found that out of the 685 MPVs consumers, 66.4% considered high prices as a negative aspect of these products.

Despite the higher prices, there is a segment of consumers and companies willing to pay more for the benefits that MPVs offer. The studies conducted by Sato et al. [27], Perez et al. [13], and Finger et al. [14] also examined the primary reasons for consumers purchasing these products, and convenience was consistently identified as the main motivating factor (88.9%, 46%, and 77.8%, respectively). In addition to being RTE, MPVs maintain the sensory and nutritional characteristics of fresh vegetables and contribute to the reduction of food waste, since the entire content is frequently used. For producers, minimal processing results in an increase in the product value and a reduction in losses during transport and storage. Moreover, by-products from minimal processing can be reused in the preparation of other foods, crop fertilization, and animal feed [16,28,29,30].

## 3. Processing of MPVs

The minimal processing of vegetables consists of several steps, including selection, cutting, washing–disinfection, rinsing, centrifuging, packaging, storage, transport, and distribution. Although some of these steps may alter the structure of vegetables, they do not alter their sensory and nutritional characteristics, resulting in fresh products that are typically RTE or RTC [10,11,16,31,32]. However, processing can increase the risk of microbial growth, since steps such as cutting and peeling may cause mechanical injury in vegetable tissues, exposing the cytoplasm and offering a nutrient source for microorganisms [9]. 

Since MPVs are usually eaten without the need for additional treatment (e.g., cooking) before consumption, minimum processing should include a step aimed at eliminating/reducing contaminants that may be present in fresh vegetables. Washing–disinfection plays an important role in this, as this step aims to remove dirt and debris, in addition to reducing the microbial load. The addition of sanitizers to washing water is important to reduce pathogenic microorganisms and especially to avoid cross-contamination between contaminated and non-contaminated products [16,33]. For instance, Maffei et al. [34] developed a quantitative microbiological risk assessment model to estimate the impact of cross-contamination during MPVs washing on the risk of salmonellosis in the population of Sao Paulo, Brazil. Their model showed that higher chlorine concentrations significantly reduced the risk of illness. Conversely, simulations using <5 ppm of free chlorine revealed that most predicted illnesses were attributed to cross-contamination, revealing the need for attention to control measures during the production of these products.

In Brazil, the use of chlorine in wash water is recommended for the disinfection of vegetables [35]. Studies conducted with MPVs processing plants and food services located in the state of Sao Paulo, Brazil, have shown that sodium dichloroisocyanurate and sodium hypochlorite (both chlorine-based compounds) are the most frequently used products for the disinfection of vegetables [17,36]. 

Chlorine and chlorine-related compounds are widely used in several countries as disinfecting agents for decontaminating fresh vegetables and MPVs, since they are low-cost, easy to apply, and have a broad spectrum of antimicrobial action [16,37]. However, their efficiency is influenced by many factors, including water temperature, pH, amount, and type of organic matter, apart from the risk of forming by-products that are harmful to human health [10,16,37,38,39]. According to the European Food Safety Authority (EFSA), the use of chlorine to disinfect vegetables is not recommended due to the risks involved. Chlorine can react with organic matter in vegetables to form harmful by-products such as trihalomethanes (THMs) and haloacetic acids (HAAs), which are potential carcinogens and can cause adverse health effects [40]. Consequently, several studies have questioned its efficacy, as its use has been insufficient in preventing previous outbreaks and recalls in the food industry [41].

Therefore, other methods for the disinfection of vegetables have been considered over the past decades, including the use of chlorine dioxide, electrolyzed water, hydrogen peroxide, ozone, organic acids, irradiation, ultrasound, ultraviolet light, and cold plasma, among others [9,16,38,42,43,44,45,46]. Other studies have explored the use of organic acids, such as acetic acid, lactic acid, and peracetic acid, as an alternative to chlorinated compounds [47,48]. Overall, these methods have shown promising results for the disinfection of vegetables, contributing to a reduction in the risk of foodborne illness by killing harmful microorganisms such as bacteria, viruses, and parasites, with the advantage of not leaving harmful residues in the water or vegetables and with a low impact on the sensory characteristics of the products.

Once MPVs are sanitized, the adoption of control measures to preserve the quality and safety of these products is recommended, as they are packaged to be protected from damage and external contamination [15]. Cold chain is essential during the storage of these products, and the recommendation is to keep them between 1 °C and 4 °C [16,32]. The combination with other techniques, such as modified atmosphere or vacuum packaging, contributes to the delay or reduction of enzymatic reactions and microbial growth during storage, thereby maintaining the organoleptic properties and extending the shelf life of these products [4,11,16,19,32,44,49]. While the modified atmosphere is created by replacing the atmospheric air inside a package with a protective gas mix (mostly oxygen, (O2), carbon dioxide (CO2), and nitrogen (N2)), vacuum packaging consists of removing all oxygen from the package, which is sealed air-tightly [11,16,19,49,50].

## 4. Microbiological Quality and Safety of MPVs

Vegetables are usually grown in open lands, and they are prone to microbial contamination at pre and post-harvesting stages. The main sources of contamination during pre-harvesting include contaminated soil, fertilizer containing raw or poorly composted animal manure, irrigation water, and the presence of domestic and wild animals in the field. During post-harvesting, the main sources include contaminated equipment, containers, and vehicles, washing and rinsing water, as well as hygiene failures while handling, transporting, and storing fresh vegetables [4,18,19,20,51,52]. Therefore, vegetables contain microorganisms coming from environmental sources, including spoilage organisms and possible foodborne pathogens. According to Beuchat [53], all types of vegetables may harbor pathogens, although *Shigella* spp., *Salmonella*, enterotoxigenic and enterohemorrhagic *Escherichia coli*, *Campylobacter* spp., *Listeria monocytogenes*, *Yersinia enterocolitica*, *Bacillus cereus*, *Clostridium botulinum*, viruses, and parasites are of the greatest public health concern.

Vegetables that undergo minimal processing go through several steps aimed at reducing the microbial load and eliminating pathogenic microorganisms. Nevertheless, failures during processing can lead to the contamination of MPVs. Contamination can arise from multiple sources, such as contaminated raw material, cross-contamination (particularly during washing), improper storage, and poor hygiene practices throughout the production chain. As can be observed in Table 2 and Table 3, several studies have evaluated the microbiological quality and safety of MPVs sold in Brazil and other countries. While some studies focus on the counts of hygiene indicator microorganisms, others include the investigation of pathogenic microorganisms, particularly *Salmonella* spp. and *L. monocytogenes*.

The presence of hygiene indicator microorganisms is crucial for assessing the microbiological quality of MPVs. Typically, these products undergo a disinfection step, and elevated microbial counts, such as generic *E. coli*, can indicate process failures. Out of the 33 studies presented in Table 2 and Table 3, a total of 21 (63.6%) reported the presence of generic *E. coli*, with counts ranging up to 8.5 logs CFU/g and 6.2 logs MPN/g. Other hygiene indicators frequently evaluated in these studies include counts of mesophilic and psychrotrophic bacteria, yeasts and molds, and *Enterobacteriaceae*, in addition to total and thermotolerant coliforms. The count ranges obtained for these microbial groups, according to the study, reached up to 10.6 logs CFU/g and 6.8 logs MPN/g. Although there is no established limit for most of these groups, it is known that high counts can indicate hygiene failures or even conditions permissive for microbial growth during storage. In addition, it can be noted that these studies focused on the determination of bacteria, with a lack of studies that evaluate the occurrence of viruses and parasites in MPVs.

Regarding pathogens, most Brazilian studies focused on the detection of *Salmonella* (50%), followed by *L. monocytogenes* (33.3%). In other countries, the search for *L. monocytogenes* is more frequent (52.4%), followed by *Salmonella* (28.6%). The prevalence of both pathogens in the MPVs samples ranged between 0.6–26.7% and 0.2–33.3% for *Salmonella* and *L. monocytogenes,* respectively. Other foodborne pathogens detected in smaller proportions (less than 30%) include *B. cereus*, *C. perfringens*, *C. sakazakii,* and *Shigella* spp. Conversely, a high prevalence of other relevant microorganisms was found in MPVs sold in some countries: *A. hydrophila* (55 and 46.1%) in Australia and Greece, respectively, *Y. enterocolitica* (59.2%) in Australia, and *S. aureus* (43.8 and 100%) in Brazil and Poland, respectively. 

Among the 33 studies cited in Table 2 and Table 3, only two reported the occurrence of pathogenic *E. coli* in MPVs, one of which was carried out in Switzerland, with a positivity of 7.7% for enteropathogenic *E. coli* (EPEC) and 0.7% for Shiga toxin-producing *E. coli* (STEC), and the other was carried out in Finland, with a positivity of 7.0% for STEC. Detecting pathogenic *E. coli* is known to be challenging due to various factors, including methodological limitations and genetic variability. Nonetheless, it is a field that deserves attention due to the increase in foodborne outbreaks caused by this bacterium. According to the WHO/FAO report entitled “Shiga toxin-producing *E. coli* (STEC) and food: attribution, characterization, and monitoring”, published in 2018, fresh produce (fruits and vegetables) accounted for the highest percentage (13%) of attributed sources of STEC globally, followed by beef (11%) and dairy products (7%) [84].

Overall, most studies cited in Table 2 and Table 3 are limited to the enumeration of hygiene indicator organisms and/or detection of bacterial pathogens using culture-dependent methods. These methods require the cultivation of microorganisms, i.e., they are usually laborious and time-consuming, and are thus capable of thoroughly depicting the actual microbial diversity present in a sample. In recent years, new techniques have emerged that enable the rapid identification of bacteria, such as mass spectrometry-based techniques. Additionally, there are approaches such as Next-Generation Sequencing (NGS) that allow for the identification of microbial communities [10,21,85,86,87]. 

Santos et al. [10] and Finger et al. [21] used the matrix-assisted laser desorption/ionization time-of-flight mass spectrometry (MALDI-TOF MS) to identify *Enterobacteriaceae* isolated from MPVs sold in Brazil. They found that the most frequent genera present in these samples were *Enterobacter* and *Pantoea*, both typical vegetable spoilers, although probably including species capable of causing opportunistic infections, mainly in immunocompromised patients. Tatsika et al. [86] used 16S rRNA gene sequencing to investigate the bacterial community composition of RTE salads at the point of consumption and the changes in bacterial diversity and composition associated with different household washing treatments. They found that Proteobacteria was the dominant phylum in the leaves of both RTE salads, with a high abundance of Enterobacteriales and Pseudomonadales, and that household treatments did not reduce the diversity of the microbial communities in these salads. Miralles et al. [85] used 16S rRNA sequencing to identify the bacterial community and the active bacterial fraction present in some of the most consumed and distributed RTE salad brands in Europe. They found *Pseudomonas* spp. as the most abundant and metabolically active bacteria in the analyzed samples. Manthou et al. [87] also used an NGS approach to decipher the bacterial communities associated with the spoilage of RTE rocket and baby spinach, and found that *Pseudomonas* spp. was the main spoilage group for both leafy vegetables.

Although there are several studies in the literature associating the occurrence of foodborne outbreaks with the consumption of contaminated fresh vegetables [16,88,89,90,91], there is a lack of studies showing this relationship with MPVs, despite the frequent occurrence of foodborne pathogens in these products. In Brazil, between 2000 and 2021, a total of 14,588 foodborne outbreaks were reported to the Brazilian Ministry of Health, including 266,247 ill individuals and 212 deaths. Among these, 153 (1%) outbreaks were associated with the consumption of contaminated vegetables, resulting in 3582 ill individuals and two deaths [91]. However, there was no information concerning whether these vegetables were consumed raw or as MPVs. Table 4 summarizes the main etiological agents and sites of occurrence of these vegetable-related outbreaks.

In the United States, data from the Centers for Disease Control and Prevention (CDC) show that 78 foodborne outbreaks linked to leafy greens were reported between 2014 and 2021. Among these, five were multistate outbreaks, of which two were linked to the consumption of packaged salads contaminated with *L. monocytogenes* (19 cases, 19 hospitalizations, and 1 death) and *Cyclospora cayetanensis* (511 cases, 24 hospitalizations, and no deaths). More recently, in 2019–2021, the CDC investigated and warned the public about nine multistate outbreaks linked to leafy greens, including six that were associated with contaminated packaged salads: two by *E. coli* O157:H7 (20 cases, 8 hospitalizations, and 1 death), two by *L. monocytogenes* (28 cases, 26 hospitalizations, and 4 deaths), one by *Salmonella* Typhimurium (31 cases, 4 hospitalizations, and no deaths), and one by *Cyclospora cayetanensis* (701 cases, 38 hospitalizations, and no deaths) [22].

Despite the diversity of microorganisms that can be found in vegetables, some microbial groups tend to be more prevalent and are more frequently involved in foodborne outbreaks. Therefore, regulatory agencies have defined microbiological criteria to guarantee the supply of safe products and to protect the health of consumers. Table 5 provides an overview of the microbiological criteria adopted in Brazil, China, the European Union (EU), and the United States (US) for assessing the microbiological quality and safety of MPVs. All entities establish guidelines for analyzing generic *E. coli* and *Salmonella* spp. as hygiene and safety indicators, respectively. For generic *E. coli*, China has the most stringent criterium (absence in 25 g), followed by the US (<3 MPN/g), Brazil (satisfactory below 10 and acceptable up to 10^2^ CFU/g), and the EU (satisfactory below 10^2^ and acceptable up to 10^3^ CFU/g). For *L. monocytogenes*, the criterium is the absence of this bacterium in 25 g (in the US) or a maximum limit of 10^2^ CFU/g (Brazil and the EU). Regarding *Salmonella*, irrespective of the country, the criterium is the absence of the pathogen in 25 g of a sample (Table 5).

The current strategies employed to mitigate bacterial contamination during the production of MPVs include the implementation of Good Agricultural Practices (GAP) during primary production and Good Handling Practices (GHP) during post-harvest stages and processing. Producers may also adopt additional approaches to ensure the quality and safety of these products, such as the application of Hazard Analysis Critical Control Point (HACCP) principles and adherence to the International Organization for Standardization (ISO) 22000 standard. These measures, combined with other relevant strategies, aim to enhance the quality and safety of these products for consumers [8,32].

## 5. Conclusions

The growing market of MPVs seems to be a trend in Brazil as these products are commonly found in major urban centers throughout the country, and the demand for them from both consumers and food companies increases. While the convenience factor contributes to increased purchases of MPVs, the higher price compared to fresh produce limits their popularity among the population. Minimal processing involves a series of carefully controlled steps to produce ready-to-eat MPVs with an extended shelf life. These steps are crucial to ensure the quality and safety of the products. In addition, data on the occurrence of hygiene indicators and pathogenic microorganisms in these products, based on the published literature, revealed that most studies focused on the detection of generic *E. coli*, *Salmonella* spp., and *L. monocytogenes*, often detected in MPVs. Finally, the records of foodborne outbreaks linked to the consumption of vegetables in Brazil highlight the importance of implementing control measures throughout the production chain to ensure the quality and safety of these products. These measures include Good Agricultural Practices in primary production and Good Handling Practices during post-harvest stages and processing, with an emphasis on the use of sanitizers during the disinfection step to eliminate microbial pathogens and prevent the occurrence of cross-contamination. Additionally, the cold chain is utilized to preserve the characteristics of these products and delay microbial growth.

The main limitations of this study were associated with the lack of available data on the international market for MPVs, particularly on prices and their relationship with fresh vegetables, which made it unfeasible to compare with the data obtained in Brazil. Additionally, it was not possible to standardize the microbiological results obtained from the studies cited in Table 2 and Table 3, as some presented a range of counts while others only presented the average count. Furthermore, it should be noted that there was no information available regarding whether the reported vegetable-associated outbreaks in Brazil were specifically linked to the consumption of fresh vegetables or MPVs. To advance research in this field, it would be valuable to conduct international collaborative studies that collect and compare data with the aim of gaining a comprehensive understanding of these products on a global level. Furthermore, conducting in-depth studies on vegetable-related outbreaks in Brazil would be of great interest to identify any potential involvement of MPVs in outbreaks.

## Figures and Tables

**Table 1:** Prices of fresh vegetables and MPVs sold in the city of Sao Paulo, Brazil.

Vegetables	Fresh Vegetables (BRL/100g)			MPVs (BRL/100g)			Price Difference
	Mean	Minimum	Maximum	Mean	Minimum	Maximum	BRL (%)
Arugula	1.88	0.78	0.79	8.72	5.32	18.87	6.84 (463.8)
Cassava	0.70	0.47	0.86	1.00	1.00	1.00	0.30 (142.8)
Escarole	0.89	0.36	1.31	7.15	4.49	9.43	6.26 (803.4)
Kale	1.58	0.59	4.00	3.88	2.00	4.50	2.30 (245.6)
Lettuce	1.05	0.68	2.39	6.12	2.99	11.32	5.07 (582.9)
Pumpkin	1.02	0.30	2.00	1.55	2.10	2.10	0.53 (152.0)
Spinach	1.83	1.66	2.00	5.65	4.61	6.00	3.82 (308.7)
Watercress	1.05	1.05	1.05	6.71	6.24	7.98	5.66 (639.0)

Values expressed in Brazilian real (BRL).

**Table 2:** Occurrence of hygiene indicators and pathogenic microorganisms in MPVs sold in Brazil.

Microorganisms	Number of Samples		Range Counts	Unit	Reference
	Total n	Positive n (%)			
Total psychrotrophic bacteria	133	133 (100)	1.0–6.0	Log CFU/g	[54]
*Enterobacteriaceae*		133 (100)	1.0 > 6.0	Log CFU/g	
Total coliforms		133 (100)	1.0–>6.0	Log CFU/g	
Thermotolerant coliforms		133 (100)	1.0–>6.0	Log CFU/g	
*Salmonella*		4 (3)	-	-	
*Listeria monocytogenes*	181	1 (0.6)	-	–	
*Listeria welshimeri*		1 (0.6)	-	-	
*Listeria innocua*		2 (1.1)	-	-	
Total mesophilic bacteria	56	56 (100)	5.7–8.2	Log CFU/g	[55]
Total psychrotrophic bacteria		56 (100)	6.9–8.2	Log CFU/g	
Thermotolerant coliforms		56 (100)	<0.5–4.0	Log MNP/g	
*Escherichia coli*		8 (28.6)	<0.5	Log MNP/g	
Oocysts of *Eimeria*	52	8 (15.3)	-	-	
Total psychrotrophic bacteria	162	157 (96.7)	7.1–9.4	Log CFU/g	[56]
Total coliforms		158 (97.5)	-	-	
Thermotolerant coliforms		107 (66)	-	-	
*Escherichia coli*		86 (53.1)	1.0–6.0	Log MNP/g	
*Listeria*		6 (3.7)	-	-	
*Listeria monocytogenes*		2 (1.2)	-	-	
*Listeria innocua*		4 (2.4)	-	-	
*Salmonella*		2 (1.2)	-	-	
Total coliforms	512	512 (100)	2.0–>6.0	Log CFU/g	[57]
*Escherichia coli*		512 (100)	2.0–5.0	Log CFU/g	
*Salmonella*		4 (0.8)	2.4–2.9	Log CFU/g	
Total mesophilic bacteria	172	172 (100)	4.0–6.8	Log CFU/g	[58]
Total coliforms		172 (100)	1.0–3.7	Log CFU/g	
*Escherichia coli*		10 (17.2)	<1.0–3.5	Log CFU/g	
*Listeria monocytogenes*		3 (1.2)	-	-	
*Salmonella*		1 (0.6)	-	-	
*Listeria monocytogenes*	512	16 (3.1)	1.0–2.4	Log CFU/g	[59]
*Cronobacter*	30	13 (43.3)	-	-	[60]
Total mesophilic bacteria	32	32 (100)	4.0–8.0	Log CFU/g	[61]
Total psychrotrophic bacteria		32 (100)	4.0–8.0	Log CFU/g	
Total coliforms		32 (100)	1.0–4.0	Log MPN/g	
Thermotolerant coliforms		32 (100)	1.0–4.0	Log MPN/g	
*Escherichia coli*		16 (50)	-	-	
*Staphylococcus aureus*		14 (43.8)	1.0–5.0	Log CFU/g	
*Salmonella*		4 (12.5)	-	-	
*Enterobacteriaceae*	100	86 (25.9)	5.2–6.8	Log MPN/g	[10]
Total coliforms		100 (100)	2.6–3.0	Log MPN/g	
Thermotolerant coliforms		20 (20)	<0.5–3.0	Log MPN/g	
*Escherichia coli*		16 (16)	<0.5–1.9	Log MPN/g	
*Salmonella*		1 (1)	-	-	
Total mesophilic bacteria	21	21 (100)	2.4–7.4	Log CFU/g	[62]
Total coliforms		9 (37.5)	0.5–>3.0	Log MPN/g	
Thermotolerant coliforms		1 (4.1)	<0.5–2.3	Log MPN/g	
*Staphylococcus*		21 (100)	<2.0–7.2	Log CFU/g	
Yeasts and molds		21 (100)	2.7–5.7	Log CFU/g	
*Enterobacteriaceae*	100	100 (100)	-	CFU/g	[21]
*Escherichia coli*		3 (3)	-	-	
*Listeria innocua*		2 (2)	-	-	
*Listeria fleischmannii*		1 (1)	-	-	
Total mesophilic bacteria	30	30 (100)	4.3–>6.3	Log CFU/g	[63]
Total coliforms		30 (100)	4.0–>6.3	Log CFU/g	
*Escherichia coli*		4 (13.3)	3.0–3.6	Log CFU/g	
Yeasts and molds		30 (100)	3.4–>6.3	Log CFU/g	

**Table 3:** Occurrence of hygiene indicators and pathogenic microorganisms in MPVs sold around the world.

Country	Microorganisms	Number of Samples		Range Counts	Unit	Reference
		Total	Positive			
		n	n (%)			
Australia	Total psychrotrophic bacteria	120	120 (100)	3.0–9.0	Log CFU/g	[64]
	*Aeromonas hydrophila or A. caviae*		66 (55)	-	-	
	*Aeromonas sobria*		14 (12.7)	-	-	
	*Listeria monocytogenes*		3 (2.5)	-	-	
	*Yersinia enterocolitica*		71 (59.2)	-	-	
Spain	Total mesophilic bacteria	236	236 (100)	4.3–8.9	Log CFU/g	[65]
	Total psychrotrophic bacteria		236 (100)	4.3–8.9	Log CFU/g	
	Lactic acid bacteria		236 (100)	<1.0–8.5	Log CFU/g	
	*Enterobacteriaceae*		236 (100)	<1.0–8.0	Log CFU/g	
	*Escherichia coli*		27 (11.4)	-	-	
	*Listeria monocytogenes*		2 (0.8)	-	-	
	*Salmonella*		4 (1.7)	-	-	
	Yeasts and molds		236 (100)	2.0–7.8	Log CFU/g	
Korea	Total mesophilic bacteria	159	159 (100)	4.2–8.9	Log CFU/g	[66]
	Total psychrotrophic bacteria		159 (100)	3.2–8.5	Log CFU/g	
	Total coliforms		159 (100)	2.2–8.2	Log CFU/g	
	*Escherichia coli*		7 (4.4)	-	-	
	*Clostridium perfringens*		6 (3.7)	-	-	
	*Salmonella*		2 (1.2)	-	-	
	Yeasts and molds		159 (100)	1.7–7.5	Log CFU/g	
Greece	Total mesophilic bacteria	26	26 (100)	5.4–8.6	Log CFU/g	[67]
	*Escherichia coli*		3 (11.5)	-	-	
	*Aeromonas*		16 (61.5)	-	-	
	*Aeromonas hydrophila*		12 (46.1)	-	-	
	*Yersinia enterocolitica*		2 (7.7)	-	-	
	Yeasts and molds		26 (100)	<3.0	Log CFU/g	
Switzerland	Total viable count	142	142 (100)	5.0–>8.0	Log CFU/g	[68]
	*Cronobacter*		2 (1.4)	-	-	
	*Escherichia coli* (EPEC)		11 (7.7)	<2.0–3.0	Log CFU/g	
	*Escherichia coli* (STEC)		1 (0.7)	<2.0	Log CFU/g	
	*Listeria monocytogenes*		5 (3.5)	<2.0	Log CFU/g	
Spain	*Listeria monocytogenes*	191	8 (4.2)	<100.0	CFU/g	[69]
Portugal	Total psychrotrophic bacteria	151	151 (100)	0.7–0.9	Log CFU/g	[70]
	*Enterobacteriaceae*		151 (100)	2.0–8.0	Log CFU/g	
	*Escherichia coli*		4 (2.6)	<1.0–2.3	Log CFU/g	
	*Listeria*		3 (2)	<1.0–2.0	Log CFU/g	
	*Listeria innocua*		2 (1.3)	2.0–2.3	Log CFU/g	
	*Listeria monocytogenes*		1 (0.7)	<2.0	Log CFU/g	
	*Aeromonas hydrophila*		11 (7.3)	3.1–5.1	Log CFU/g	
	*Bacillus cereus*	66	15 (22.7)	<2.0–3.2	Log CFU/g	
France	*Clostridium difficile*	104	3 (2.9)	-	-	[71]
Croatia	*Listeria monocytogenes*	100	1 (1)	1.8	Log CFU/g	[72]
	*Listeria*		20 (20)	-	-	
Iran	Total mesophilic bacteria	32	32 (100)	5.3–7.5	Log CFU/g	[73]
	Total coliforms		28 (87.5)	ND *–5.5	Log CFU/g	
	Thermotolerant coliforms		11 (34.4)	-	-	
	*Escherichia coli*		3 (9.4)	-	-	
	Yeasts and molds		32 (100)	5.4–7.6	Log CFU/g	
Mexico	Total mesophilic bacteria	100	100 (100)	3.0–6.6	Log CFU/g	[74]
	Total coliforms		96 (100)	<0.5–>3.0	Log NMP/g	
	Thermotolerant coliforms		32 (32)	<0.5–>3.0	Log NMP/g	
	Nontuberculous mycobacteria		7 (7)	-	-	
Turkey	Total psychrotrophic bacteria	261	235 (90)	2.0–> 6.0	Log CFU/g	[75]
	Total coliforms		155 (59.3)	>0.5	Log NMP/g	
	*Escherichia coli*		10 (3.8)	>0.5	Log NMP/g	
	*Listeria monocytogenes*		15 (5.7)	-	-	
	*Listeria ivanovi*		14 (5.3)	-	-	
	*Listeria grayi*		21 (8)	-	-	
	*Listeria welshimeri*		23 (8.8)	-	-	
	*Salmonella*		21 (8)	-	-	
Finland	Total mesophilic bacteria	100	100 (100)	6.2–10.6	Log CFU/g	[76]
	Total coliforms		100 (100)	4.2–8.3	Log CFU/g	
	*Escherichia coli*		15 (15)	-	-	
	*Escherichia coli* (STEC)		7 (7)	-	-	
	*Listeria*		4 (4)	-	-	
	*Listeria monocytogenes*		2 (2)	-	-	
	*Yersinia*		33 (33)	-	-	
	*Yersinia enterocolitica*		3 (3)	-	-	
	*Salmonella*		2 (2)	-	-	
Egypt	Total mesophilic bacteria	50	10 (35.7)	3.8–9.4	Log CFU/g	[77]
	Total coliforms		33 (66)	-	-	
	Thermotolerant coliforms		33 (66)	-	-	
	*Escherichia coli*		4 (18.2)	-	-	
Poland	Total mesophilic bacteria	20	20 (100)	5.6–7.6	Log CFU/g	[78]
	*Cronobacter*		6 (35)	-	-	
	*Cronobacter sakazakii*		3 (15)	-	-	
Italy	Total mesophilic bacteria	78	78 (100)	6.0–9.2	Log CFU/g	[79]
Ecuador	Total mesophilic bacteria	60	60 (100)	4.5–7.8	Log CFU/g	[80]
	Total coliforms		60 (100)	0.4–>5.0	Log MNP/g	
	*Escherichia coli*		13 (21.7)	<0.8	Log MNP/g	
Canada	*Listeria monocytogenes*	5379	13 (0.2)	-	-	[46]
Iran	*Escherichia coli*	92	28 (30.4)	-	-	[81]
	*Clostridium perfringens*		8 (8.7)	-	-	
	*Bacillus cereus*		10 (10.9)	-	-	
	*Listeria monocytogenes*		4 (4.3)	-	-	
	*Staphylococcus aureus*		18 (19.6)	-	-	
	*Pseudomonas aeruginosa*		4 (4.3)	-	-	
	*Shigella*		2 (2.2)	-	-	
	*Salmonella*		3 (3.3)	-	-	
Argentina	Total coliforms	60	60 (100)	1.3–3.3	Log MPN/g	[82]
	Thermotolerant coliforms		60 (100)	0.3–1.9	Log MPN/g	
	*Escherichia coli*		15 (25)	3.4–8.4	Log CFU/g	
	*Staphylococcus aureus*		3 (5)	-	-	
Poland	Total mesophilic bacteria	30	30 (100)	2.3–9.3	Log CFU/g	[83]
	*Enterobacteriaceae*		30 (100)	<1.0–7.4	Log CFU/g	
	*Escherichia coli*		30 (100)	<1.0–5.5	Log CFU/g	
	*Staphylococcus aureus*		30 (100)	<1.0–3.5	Log CFU/g	
	Lactic acid bacteria		30 (100)	<1.0–8.4	Log CFU/g	
	*Listeria monocytogenes*		10 (33.3)	-	-	
	*Salmonella*		8 (26.7)	-	-	
	Yeasts and molds		30 (100)	<1.0–7.0	Log CFU/g	

* ND—not detected.

**Table 4:** Etiological agents and sites of occurrence of foodborne outbreaks linked to vegetables in Brazil between 2000 and 2021.

Etiological Agents	Outbreaks		Sick Individuals		Dead Individuals
	n	%	n	%	n
Not identified	39	25.5	703	19.6	1
* Escherichia coli * *	27	17.6	752	21	0
* Salmonella * spp.	25	16.3	681	19	0
* Bacillus cereus *	20	13.1	543	15.2	0
* Staphylococcus aureus *	14	9.2	515	14.4	0
Others	28	18.3	388	10.8	1
Total	153	100	3582	100	2
Sites of occurrence					
Restaurants/bakeries	31	20.3	270	7.5	0
Homes	31	20.3	222	6.2	2
Other institutions (accommodation facilities, workplace)	29	19	1271	35.5	0
Others	62	40.4	1819	50.8	0
Total	153	100	3582	100	2

Source: [92]. * No information is given on whether the outbreak was caused by a pathogenic pathovar of *E. coli*.

**Table 5:** Microbiological criteria for MPVs around the world.

Source	Criteria	Guidelines									
		*Bacillus cereus*	*Campylobacter* spp.	*Clostridium perfringens*	*Escherichia coli*	*Listeria monocytogenes*	*Salmonella* spp.	*Staphylococcus aureus*	*Vibrio cholerae*	*Vibrio parahaemolyticus*	Yeasts and Molds
Brazilian Ministry of Health [93]	Satisfactory	N/A	N/A	N/A	10	10^2^	Abs/25 g	N/A	N/A	N/A	N/A
	Acceptable	N/A	N/A	N/A	10^2^	N/A	N/A	N/A	N/A	N/A	N/A
Centre for Food Safety China [94]	Satisfactory	<10^3^	Abs/25 g	<10	Abs/25 g	Abs/25 g	Abs/25 g	<20	Abs/25 g	<20	Abs/25 g
	Acceptable	10^3^–≤10^5^	N/A	10–≤10^4^	N/A	N/A	N/A	20–≤10^4^	N/A	20–≤10^3^	N/A
European Union [95]	Satisfactory	N/A	N/A	N/A	10^2^	10^2^	Abs/25 g	N/A	N/A	N/A	N/A
	Acceptable	N/A	N/A	N/A	10^3^	N/A	N/A	N/A	N/A	N/A	N/A
Food and Drug Administration USA [96]	Satisfactory	N/A	N/A	N/A	<3 *	N/A	Abs/25 g	10	N/A	N/A	10^2^
	Acceptable	N/A	N/A	N/A	N/A	Abs/25 g	N/A	N/A	N/A	N/A	10^4^

Satisfactory: the microbiological status of the food sample is satisfactory. Acceptable: the microbiological status of the food sample is less than satisfactory but still acceptable for consumption. Values expressed as Colony Forming Units per gram (CFU/g) or * Most Probable Number per gram (MPN/g). Abs/25 g: absence in 25 g. N/A: not applicable.

## References

[1] Brasil. Ministério da Saúde, Secretaria de Atenção à Saúde, Departamento de Atenção Básica (2014). Guia Alimentar para a População Brasileira.

[2] Saini R.K., Ko E.Y., Keum Y.S. (2017). Minimally processed ready-to-eat baby-leaf vegetables: Production, processing, storage, microbial safety, and nutritional potential. Food Rev. Int..

[3] Maffei D.F., Silveira M.A., Silva M.B.R.D., Moreira D.A., Lourenço F.R., Schaffner D.W., Franco B.D.G.M. (2020). Consumption data and consumer handling practices of leafy greens in the city of São Paulo, Brazil: Useful information for quantitative microbiological consumer phase risk assessments. Food Prot. Trends.

[4] Mostafidi M., Sanjabi M.R., Shirkhan F., Zahedi M.T. (2020). A review of recent trends in the development of the microbial safety of fruits and vegetables. Trends Food Sci. Technol..

[5] FAO (Food and Agriculture Organization of the United Nations) (2021). Fruit and Vegetables—Your Dietary Essentials. The International Year of Fruits and Vegetables, Background Paper. Rome..

[6] Alvarenga A.L.B., Toledo J.C.D., Paulillo L.F.D.O. (2014). Qualidade e segurança de vegetais minimamente processados: Proposta de estruturas de governança entre os agentes da cadeia e os sinais da qualidade. Gest. Prod..

[7] Vieira S.L.V., da Silva I.C.P. (2017). Alimentos minimamente processados: Novo perfil de escolha do consumidor. Arq. MUDI.

[8] Maldonade I.R., Ginani V.C., Riquette R.F.R., Gurgel-Gonçalves R., Mendes V.S., Machado E.R. (2019). Good manufacturing practices of minimally processed vegetables reduce contamination with pathogenic microorganisms. Rev. Inst. Med. Trop. São Paulo.

[9] de Corato U. (2020). Improving the shelf-life and quality of fresh and minimally-processed fruits and vegetables for a modern food industry: A comprehensive critical review from the traditional technologies into the most promising advancements. Crit. Rev. Food Sci. Nutr..

[10] Santos T.S., Campos F.B., Padovani N.F.D.A., Dias M., Mendes M.A., Maffei D.F. (2020). Assessment of the microbiological quality and safety of minimally processed vegetables sold in Piracicaba, SP, Brazil. Lett. Appl. Microbiol..

[11] Velderrain-Rodríguez G.R., López-Gámez G.M., Domínguez-Avila J.A., González-Aguilar G.A., Soliva-Fortuny R., Ayala-Zavala J.F. (2019). Minimal processing. Postharvest Technology of Perishable Horticultural Commodities.

[12] de Corato U., Cancellara F.A. (2019). Measures, technologies, and incentives for cleaning the minimally processed fruits and vegetables supply chain in the Italian food industry. J. Clean. Prod..

[13] Perez R., Ramos A.M., Binoti M.L., Sousa P.H.M.D., Machado G.D.M., Cruz I.B. (2008). Perfil dos consumidores de hortaliças minimamente processadas de Belo Horizonte. Hortic. Bras..

[14] Finger J.A.F.F., Costa D.A., Alves V.F., Baroni W.S.G.V., Malheiros P.S., Alves E.A., Maffei D.F., Pinto U.M. (2023). Minimally Processed Vegetables: Consumer Profile, Consumption Habits, and Perceptions of Microbiological Risk. Food Prot. Trends.

[15] Moretti C.L. (2007). Manual de Processamento Mínimo de Frutas e Hortaliças.

[16] Sant’Anna P.B., Franco B.D.G.M., Maffei D.F. (2020). Microbiological safety of ready-to-eat minimally processed vegetables in Brazil: An overview. J. Sci. Food Agric..

[17] Maffei D.F., Alvarenga V.O., Sant’Ana A.S., Franco B.D.G.M. (2016). Assessing the effect of washing practices employed in Brazilian processing plants on the quality of ready-to-eat vegetables. LWT Food Sci. Technol..

[18] Fröhling A., Rademacher A., Rumpold B., Klocke M., Schlüter O. (2018). Screening of microbial communities associated with endive lettuce during postharvest processing on industrial scale. Heliyon.

[19] Mir S.A., Shah M.A., Mir M.M., Dar B.N., Greiner R., Roohinejad S. (2018). Microbiological contamination of ready-to-eat vegetable salads in developing countries and potential solutions in the supply chain to control microbial pathogens. Food Control..

[20] Machado-Moreira B., Richards K., Brennan F., Abram F., Burgess C.M. (2019). Microbial contamination of fresh produce: What, where, and how?. Compr. Rev. Food Sci. Food Saf..

[21] Finger J.A.F.F., Maffei D.F., Dias M., Mendes M.A., Pinto U.M. (2021). Microbiological quality and safety of minimally processed parsley (*Petroselinum crispum*) sold in food markets, southeastern Brazil. J. Appl. Microbiol..

[22] CDC (Centers for Disease Control and Prevention) (2022). Center of Disease Control. Food Safety. Foodborne Outbreaks. https://www.cdc.gov/foodsafety/outbreaks/index.html.

[23] Paoletti F., Raffo A. (2022). Fresh-Cut Vegetables Processing: Environmental Sustainability and Food Safety Issues in a Comprehensive Perspective. Front. Sustain. Food Syst..

[24] Nascimento K.D.O., Augusta I.M., da Rocha Rodrigues N., Pires T., Batista E., Júnior J.L.B., Barbosa M.I.M.J. (2014). Alimentos minimamente processados: Uma tendência de mercado. Acta Tecnol..

[25] Costa D.A., Finger J.A.F.F., Alves V.F., Baroni W.S.G.V., Malheiros P.S., Alves E.A., Maffei D.F., Pinto U.M. (2021). Minimally Processed Vegetables: Consumer Profile, Consumption Habits, Perceptions of Microbiological Risk and Labeling. In Anais do 31° Congresso Brasileiro de Microbiologia: Brasil. https://sbmicrobiologia.org.br/31cbm-anais/lista_area_01.htm.

[26] Embrapa (Empresa Brasileira de Pesquisa Agropecuária Agroindústria Tropical) (2011). Processamento Mínimo de Frutas E Hortaliças: Tecnologia, Qualidade E Sistemas de Embalagem/Coordenador, Sergio Agostinho Cenci.

[27] Sato G.S., Martins V.A., Bueno C.R.F. (2007). Análise exploratória do perfil do consumidor de produtos minimamente processados na cidade de São Paulo. Inf. Econômicas.

[28] Degiovanni G.C., Japur C.C., Sanches A.P.L.M., Mattos C.H.P.D.S., Martins L.D.S., Reis C.V.D., Vieira M.N.C.M. (2010). Hortaliças in natura ou minimamente processadas em unidades de alimentação e nutrição: Quais aspectos devem ser considerados na sua aquisição?. Rev. Nutr..

[29] Pena F.L., Paulo K.H., Soragni L., Duarte L.T., Antunes A.E.C. (2015). Avaliação microbiológica de hortaliças minimamente processadas disponíveis no mercado e servidas em redes de fast-food e em unidades de alimentação e nutrição nas cidades de Limeira e Campinas, São Paulo, Brasil. Rev. Segur. Aliment. Nutr..

[30] Melo V.T.P., Strasburg V.J. (2020). Geração de resíduos na aquisição de vegetais in natura e minimamente processados por serviço de nutrição e dietética de um hospital público. Braz. J. Food Technol..

[31] Bansal V., Siddiqui M.W., Rahman M.S., Siddiqui M., Rahman M. (2015). Minimally processed foods: Overview. Minimally Processed Foods: Technologies for Safety, Quality, and Convenience.

[32] Castro-Ibáñez I., Gil M.I., Allende A. (2017). Ready-to-eat vegetables: Current problems and potential solutions to reduce microbial risk in the production chain. LWT Food Sci. Technol..

[33] López-Gálvez F., Gil M.I., Truchado P., Selma M.V., Allende A. (2010). Cross-contamination of fresh-cut lettuce after a short-term exposure during pre-washing cannot be controlled after subsequent washing with chlorine dioxide or sodium hypochlorite. Food Microbiol..

[34] Maffei D.F., Sant’Ana A.S., Franco B.D.G.M., Schaffner D.W. (2017). Quantitative assessment of the impact of cross-contamination during the washing step of ready-to-eat leafy greens on the risk of illness caused by *Salmonella*. Food Res. Int..

[35] Paulo S. (2013). Secretária do Estado da Saúde. Portaria CVS 5, de 09 de abril de 2013. Aprova o Regulamento Técnico Sobre Boas Práticas Para Estabelecimentos Comerciais de Alimentos e Para Serviços de Alimentação, e o Roteiro de Inspeção, Anexo.

[36] Ferreira M.R., Santos T.S.D., Maffei D.F. (2021). Assessing Brazilian food establishments’ hygienic handling of leafy vegetables and their microbiological quality. Acta Aliment..

[37] Lee W.N., Huang C.H., Zhu G. (2019). Analytical methods for conventional and emerging disinfection by-products in fresh-cut produce. Food Chem..

[38] Joshi K., Mahendran R., Alagusundaram K., Norton T., Tiwari B.K. (2013). Novel disinfectants for fresh produce. Trends Food Sci. Technol..

[39] Feliziani E., Lichter A., Smilanick J.L., Ippolito A. (2016). Disinfecting agents for controlling fruit and vegetable diseases after harvest. Postharvest Biol. Technol..

[40] Gadelha J.R., Allende A., López-Gálvez F., Fernández P., Gil M.I., Egea J.A. (2019). Chemical risks associated with ready-to-eat vegetables: Quantitative analysis to estimate formation and/or accumulation of disinfection byproducts during washing. EFSA J..

[41] Yoon J.H., Lee S.Y. (2018). Comparison of the effectiveness of decontaminating strategies for fresh fruits and vegetables and related limitations. Crit. Rev. Food Sci. Nutr..

[42] Meireles A., Giaouris E., Simões M. (2016). Alternative disinfection methods to chlorine for use in the fresh-cut industry. Food Res. Int..

[43] Fan X., Huang R., Chen H. (2017). Application of ultraviolet C technology for surface decontamination of fresh produce. Trends Food Sci. Technol..

[44] Ma L., Zhang M., Bhandari B., Gao Z. (2017). Recent developments in novel shelf-life extension technologies of fresh-cut fruits and vegetables. Trends Food Sci. Technol..

[45] Balbinot Filho C.A., Borges C.D. (2020). Efeitos da radiação UV-C em alface e maçã minimamente processadas: Uma revisão. Braz. J. Food Technol..

[46] Zhang H., Tikekar R.V., Ding Q., Gilbert A.R., Wimsatt S.T. (2020). Inactivation of foodborne pathogens by the synergistic combinations of food processing technologies and food-grade compounds. Compr. Rev. Food Sci. Food Saf..

[47] Lepaus B.M., Rocha J.S., de São José J.F.B. (2020). Organic Acids and Hydrogen Peroxide Can Replace Chlorinated Compounds as Sanitizers on Strawberries, Cucumbers and Rocket Leaves. Food Sci. Technol..

[48] Lippman B., Yao S., Huang R., Chen H. (2020). Evaluation of the Combined Treatment of Ultraviolet Light and Peracetic Acid as an Alternative to Chlorine Washing for Lettuce Decontamination. Int. J. Food Microbiol..

[49] Onwude D.I., Chen G., Eke-Emezie N., Kabutey A., Khaled A.Y., Sturm B. (2020). Recent advances in reducing food losses in the supply chain of fresh agricultural produce. Processes.

[50] Denoya G.I., Vaudagna S.R., Polenta G. (2015). Effect of high pressure processing and vacuum packaging on the preservation of fresh-cut peaches. LWT Food Sci. Technol..

[51] Gil M.I., Selma M.V., Suslow T., Jacxsens L., Uyttendaele M., Allende A. (2015). Pre-and postharvest preventive measures and intervention strategies to control microbial food safety hazards of fresh leafy vegetables. Crit. Rev. Food Sci. Nutr..

[52] Maffei D.F., Batalha E.Y., Landgraf M., Schaffner D.W., Franco B.D.G.M. (2016). Microbiology of organic and conventionally grown fresh produce. Braz. J. Microbiol..

[53] Beuchat L.R. (2002). Ecological factors influencing survival and growth of human pathogens on raw fruits and vegetables. Microbes Infect..

[54] Fröder H., Martins C.G., de Souza K.L.O., Landgraf M., Franco B.D.G.M., Destro M.T. (2007). Minimally processed vegetable salads: Microbial quality evaluation. J. Food Prot..

[55] Silva S.R., Verdin S.E.F., Pereira D.C., Schatkoski A.M., Rott M.B., Corção G. (2007). Microbiological quality of minimally processed vegetables sold in Porto Alegre, Brazil. Braz. J. Microbiol..

[56] de Oliveira M.A., de Souza V.M., Bergamini A.M.M., de Martinis E.C.P. (2011). Microbiological quality of ready-to-eat minimally processed vegetables consumed in Brazil. Food Control..

[57] Sant’Ana A.S., Landgraf M., Destro M.T., Franco B.D.G.M. (2011). Prevalence and counts of *Salmonella* spp. in minimally processed vegetables in São Paulo, Brazil. Food Microbiol..

[58] Maistro L.C., Miya N.T.N., Sant’Ana A.S., Pereira J.L. (2012). Microbiological quality and safety of minimally processed vegetables marketed in Campinas, SP–Brazil, as assessed by traditional and alternative methods. Food Control..

[59] Sant’Ana A.S., Igarashi M.C., Landgraf M., Destro M.T., Franco B.D.G.M. (2012). Prevalence, populations and pheno-and genotypic characteristics of *Listeria monocytogenes* isolated from ready-to-eat vegetables marketed in São Paulo, Brazil. Int. J. Food Microbiol..

[60] Vasconcellos L., Carvalho C.T., Tavares R.O., Medeiros V.M., Rosas C.O., Silva J.N., Lopes S.M.D.R., Forsythe S.J., Brandão M.L.L. (2018). Isolation, molecular and phenotypic characterization of *Cronobacter* spp. in ready-to-eat salads and foods from Japanese cuisine commercialized in Brazil. Food Res. Int..

[61] Cruz M.R.G.D., Leite Y.J.B.D.S., Marques J.D.L., Pavelquesi S.L.S., Oliveira L.R.D.A., Silva I.C.R.D., Orsi D.C. (2019). Microbiological quality of minimally processed vegetables commercialized in Brasilia, DF, Brazil. Food Sci. Technol..

[62] Schuh V., Schuh J., Fronza N., Foralosso F.B., Verruck S., Vargas Junior A., Silveira S.M.D. (2020). Evaluation of the microbiological quality of minimally processed vegetables. Food Sci. Technol..

[63] Santos L.S., Silva L.V., Lepaus B.M., São José J.F.B. (2021). Microbial quality and labeling of minimally processed fruits and vegetables. Biosci. J..

[64] Szabo E.A., Scurrah K.J., Burrows J.M. (2000). Survey for psychrotrophic bacterial pathogens in minimally processed lettuce. Lett. Appl. Microbiol..

[65] Abadias M., Usall J., Anguera M., Solsona C., Viñas I. (2008). Microbiological quality of fresh, minimally-processed fruit and vegetables, and sprouts from retail establishments. Int. J. Food Microbiol..

[66] Seo Y.H., Jang J.H., Moon K.D. (2010). Microbial evaluation of minimally processed vegetables and sprouts produced in Seoul, Korea. Food Sci. Biotechnol..

[67] Xanthopoulos V., Tzanetakis N., Litopoulou-Tzanetaki E. (2010). Occurrence and characterization of *Aeromonas hydrophila* and *Yersinia enterocolitica* in minimally processed fresh vegetable salads. Food Control..

[68] Althaus D., Hofer E., Corti S., Julmi A., Stephan R. (2012). Bacteriological survey of ready-to-eat lettuce, fresh-cut fruit, and sprouts collected from the Swiss market. J. Food Prot..

[69] Moreno Y., Sánchez-Contreras J., Montes R.M., García-Hernández J., Ballesteros L., Ferrús M.A. (2012). Detection and enumeration of viable *Listeria monocytogenes* cells from ready-to-eat and processed vegetable foods by culture and DVC-FISH. Food Control..

[70] Santos M.I., Cavaco A., Gouveia J., Novais M.R., Nogueira P.J., Pedroso L., Ferreira M.A.S.S. (2012). Evaluation of minimally processed salads commercialized in Portugal. Food Control.

[71] Eckert C., Burghoffer B., Barbut F. (2013). Contamination of ready-to-eat raw vegetables with *Clostridium difficile* in France. J. Med. Microbiol..

[72] Kovačević M., Burazin J., Pavlović H., Kopjar M., Piližota V. (2013). Prevalence and level of *Listeria monocytogenes* and other *Listeria* sp. in ready-to-eat minimally processed and refrigerated vegetables. World J. Microbiol. Biotechnol..

[73] Jeddi M.Z., Yunesian M., Gorji M.E.H., Noori N., Pourmand M.R., Khaniki G.R.J. (2014). Microbial evaluation of fresh, minimally-processed vegetables and bagged sprouts from chain supermarkets. J. Health Popul. Nutr..

[74] Cerna-Cortes J.F., Leon-Montes N., Cortes-Cueto A.L., Salas-Rangel L.P., Helguera-Repetto A.C., Lopez-Hernandez D., Gonzalez-y-Merchand J.A. (2015). Microbiological quality of ready-to-eat vegetables collected in Mexico City: Occurrence of aerobic-mesophilic bacteria, fecal coliforms, and potentially pathogenic nontuberculous mycobacteria. BioMed Res. Int..

[75] Gurler Z., Pamuk S., Yildirim Y., Ertas N. (2015). The microbiological quality of ready-to-eat salads in Turkey: A focus on *Salmonella* spp. and *Listeria monocytogenes*. Int. J. Food Microbiol..

[76] Nousiainen L.L., Joutsen S., Lunden J., Hänninen M.L., Fredriksson-Ahomaa M. (2016). Bacterial quality and safety of packaged fresh leafy vegetables at the retail level in Finland. Int. J. Food Microbiol..

[77] Abaza A. (2017). Bacteriological assessment of some vegetables and ready-to-eat salads in Alexandria Egypt. J. Egypt Public Health Assoc..

[78] Berthold-Pluta A., Garbowska M., Stefańska I., Pluta A. (2017). Microbiological quality of selected ready-to-eat leaf vegetables, sprouts and non-pasteurized fresh fruit-vegetable juices including the presence of *Cronobacter* spp.. Food Microbiol..

[79] Bencardino D., Vitali L.A., Petrelli D. (2018). Microbiological evaluation of ready-to-eat iceberg lettuce during shelf-life and effectiveness of household washing methods. Ital. J. Food Saf..

[80] Hualpa D., Toledo Z., Meneses M.A., Feng P. (2018). Microbiological Quality of Minimally Processed, Ready-to-Eat, Vegetables in Loja, Ecuador. Rev. Politec..

[81] Azimirad M., Nadalian B., Alavifard H., Panirani S.N., Bonab S.M.V., Azimirad F., Gholami F., Jabbari P., Yadegar A., Busani L. (2021). Microbiological survey and occurrence of bacterial foodborne pathogens in raw and ready-to-eat green leafy vegetables marketed in Tehran, Iran. Int. J. Hyg. Environ. Health.

[82] Baraquet M.L., Camiletti O.F., Moretti C.I., Rodríguez L.E., Vázquez C., Oberto M.G. (2021). Microbiological Status and Quality Traits of Ready-to-Eat Minimally Processed Vegetables Sold in Córdoba, Argentina. J. Food Qual. Hazards Control..

[83] Łepecka A., Zielińska D., Szymański P., Buras I., Kołożyn-Krajewska D. (2022). Assessment of the Microbiological Quality of Ready-to-Eat Salads—Are There Any Reasons for Concern about Public Health?. Int. J. Environ. Res. Public Health.

[84] WHO (World Health Organization), FAO (Food and Agriculture Organization of the United Nations) (2018). Shiga Toxin-Producing Escherichia coli (STEC) and Food: Attribution, Characterization, and Monitoring: Report.

[85] Miralles M.M., Maestre-Carballa L., Lluesma-Gomez M., Martinez-Garcia M. (2019). High-throughput 16S rRNA sequencing to assess potentially active bacteria and foodborne pathogens: A case example in ready-to-eat food. Foods.

[86] Tatsika S., Karamanoli K., Karayanni H., Genitsaris S. (2019). Metagenomic characterization of bacterial communities on ready-to-eat vegetables and effects of household washing on their diversity and composition. Pathogens.

[87] Manthou E., Coeuret G., Chaillou S., Nychas G.J.E. (2022). Metagenetic characterization of bacterial communities associated with ready-to-eat leafy vegetables and study of temperature effect on their composition during storage. Food Res. Int..

[88] Jung Y., Jang H., Matthews K.R. (2014). Effect of the food production chain from farm practices to vegetable processing on outbreak incidence. J. Microbial. Biotechnol..

[89] Callejón R.M., Rodríguez-Naranjo M.I., Ubeda C., Hornedo-Ortega R., Garcia-Parrilla M.C., Troncoso A.M. (2015). Reported foodborne outbreaks due to fresh produce in the United States and European Union: Trends and causes. Foodborne Pathog. Dis..

[90] Garner D., Kathariou S. (2016). Fresh produce-associated listeriosis outbreaks, sources of concern, teachable moments, and insights. J. Food Prot..

[91] Elias S.O., Decol L.T., Tondo E.C. (2018). Foodborne outbreaks in Brazil associated with fruits and vegetables: 2008 through 2014. Food Qual. Saf..

[92] Ministério da Saúde, Secretaria de Vigilância em Saúde, Sistema de Informação de Agravos de Notificação (2022). Situação Epidemiológica—Doenças Transmitidas Por Alimentos.

[93] Ministério da Saúde, Agência Nacional de Vigilância Sanitária (2022). Instrução Normativa n 161, de 01 de Julho de 2022.

[94] CFS (Centre for Food Safety) (2014). Microbiological Guidelines for Food (For Ready-To-Eat Food in General and Specific Food Items). In Food and Environmental Hygiene Department, Hong Kong. https://www.cfs.gov.hk/english/food_leg/files/food_leg_Microbiological_Guidelines_for_Food_e.pdf.

[95] EU (European Union) (2005). Commission Regulation (EC) No 2073/2005 of 15 November 2005 on microbiological criteria for foodstuffs. Off. J. Eur. Union.

[96] FDA (Food and Drug Administration) (2013). FDA Circular 2013-010-Food and Drug Administration Philippines. https://members.wto.org/crnattachments/2021/TBT/PHL/21_3930_00_e.pdf.

